# Twelve-Month Outcomes of Standalone Travoprost Intracameral Implant in Glaucoma or Ocular Hypertension

**DOI:** 10.3390/life16040614

**Published:** 2026-04-07

**Authors:** Savak Teymoorian, Jasmin Kaur, Dana M. Hornbeak, Erik Barr

**Affiliations:** 1 Harvard Eye Associates, Laguna Hills, CA 92653, USA; jkaur@harvardeye.com; 2 Glaukos Corporation, Aliso Viejo, CA 92656, USA; dhornbeak@glaukos.com (D.M.H.); ebarr@glaukos.com (E.B.)

**Keywords:** iDose TR, prostaglandin analogs, travoprost implant, pharmaceutical, glaucoma

## Abstract

This retrospective study evaluated real-world outcomes of standalone iDose TR intracameral travoprost implant administration. Sixty-five consecutive standalone iDose TR implantations performed by a single surgeon were analyzed. Patients were pseudophakic, had a diagnosis of open-angle glaucoma (OAG) or ocular hypertension (OHT), and had a history of a prior non-filtering glaucoma procedure (e.g., selective laser trabeculoplasty [SLT] or bimatoprost intracameral implant) performed beyond the preceding 6 months. Intraocular pressure (IOP) and medications were measured for 12 months postoperatively. Subgroup analysis was stratified by history of SLT treatment and glaucoma severity. If target IOP was not attained, secondary minimally invasive glaucoma surgery was performed instead of reinitiating or adding medication, according to the surgeon’s standard practice. The analysis was by intention to treat. At 12 months, mean IOP reduced significantly to 14.0 ± 2.9 mmHg from a baseline of 20.0 ± 4.0 mmHg (−28%, *p* < 0.001). Eyes with IOPs ≤ 18, ≤15, and ≤12 mmHg increased significantly vs. baseline (36.9% to 92.3%, 10.8% to 73.8%, and 3.1% to 35.4%, respectively; all *p* < 0.001), and 89.2% of the eyes were medication-free vs. 87.7% preoperatively. Mean 12-month IOP reduction showed nonsignificant differences between eyes with or without prior SLT (−26% and −31%, respectively; *p* = 0.907) and among mild/OHT, moderate, or severe glaucoma eyes (−28%, −23%, and −34%, respectively; *p* = 0.085). Postoperatively, one case each of transient corneal edema and retinal edema were observed, which self-resolved without sequelae. Thus, standalone travoprost implant administration significantly reduced IOP over 12 months in OAG and OHT, while maintaining a low medication burden. Similar IOP reductions were observed regardless of prior SLT treatment and glaucoma severity.

## 1. Introduction

Glaucoma is a progressive optic neuropathy that involves the gradual death of retinal ganglion cells, and the only proven strategy to avoid glaucomatous blindness is a reduction in intraocular pressure (IOP) [1]. Topical medications are the most common first-line therapy in glaucoma, but IOP control can be suboptimal due to the well-known problem of patient noncompliance. Up to 80% patients do not adhere to their prescribed treatment regimens [2] due to several factors, including forgetfulness, complex regimens with multiple drops, and difficulty self-administering eyedrops [3]. Additionally, topical medications cannot consistently maintain IOP reduction over a 24 h period, leading to wider IOP fluctuations as compared to glaucoma surgery [4,5]. Both medication nonadherence and IOP fluctuations have been associated with a higher risk of glaucomatous vision loss [6,7], limiting the long-term utility of topical pharmacotherapy.

The recognition of these key limitations has led to a shift away from the traditional topical medication-first paradigm to earlier interventions with minimally invasive modalities such as procedural pharmaceuticals and minimally invasive glaucoma surgery (MIGS). This approach, known as interventional glaucoma, aims to proactively prevent vision loss by ensuring steady 24 h IOP control without relying on patient adherence [8]. In particular, procedural pharmaceuticals deliver sustained and consistent medication levels while removing the burden of self-dosing. The sustained-release implants approved by the US Food and Drug Administration (FDA) are the bimatoprost intracameral implant Durysta (Allergan, Irvine, CA, USA) and the travoprost intracameral implant iDose TR (Glaukos Corporation, Aliso Viejo, CA, USA).

The travoprost intracameral implant was compared with twice-daily timolol drops in Phase 2 and Phase 3 trials conducted on patients with mild to moderate open-angle glaucoma (OAG) and ocular hypertension (OHT). In the Phase 2 trial, the iDose TR implant showed significant IOP reduction up to 36 months postoperatively (*p* < 0.0001), and 69% of patients were well-controlled on the same or fewer topical medications relative to baseline vs. 45% of patients in the timolol group (*p* = 0.0548) [9]. In vivo human pharmacokinetic studies have confirmed this longevity, showing that the travoprost implant provides consistent drug concentrations above the minimum efficacious levels up to 24 months, with the lifetime drug elution estimated to continue up to 36.5 months [10]. In the Phase 3 trials, the iDose TR demonstrated non-inferiority to timolol over 12 months postoperatively, with 93% of eyes on the same or fewer medications compared to baseline vs. 67% of timolol eyes (*p* < 0.0001) [11]. The implant showed favorable safety across all trials, with no instances of corneal adverse events, prostaglandin-associated periorbitopathy, or periocular hyperpigmentation.

Previously, we reported the first real-world outcomes of the travoprost intracameral implant in a retrospective 3-month study [12]. Following standalone iDose TR implantation in pseudophakic patients with OAG or OHT, clinically and statistically significant reductions in mean IOP and medication burden were observed for 3 months postoperatively (33% and 100% reductions from 19.6 ± 3.8 mmHg and 0.28 ± 0.71 medications at baseline, respectively; *p* < 0.01 for both), and all eyes were medication-free at 3 months.

In the present study, we evaluate the 12-month efficacy and safety of a single standalone travoprost intracameral implant administration in a real-world setting.

## 2. Materials and Methods

### 2.1. Study Design and Participants

This retrospective consecutive case series included all cases of standalone iDose TR implantation performed by a single surgeon in a single center (Harvard Eye Associates, Laguna Hills, CA, USA) from February 2024 to April 2024. The eligibility criteria were age over 18 years, pseudophakia, a diagnosis of OAG or OHT, and a need for reduction in IOP and/or medication burden as determined by the treating ophthalmologist. Any glaucoma severity could be included. Exclusion criteria were abnormalities of the anterior chamber angle precluding implant placement, active intraocular inflammation, a history of filtration surgery in the past 12 months, or a history of non-filtering procedure (e.g., selective laser trabeculoplasty [SLT], procedural pharmaceuticals, or MIGS) in the 6 months prior to iDose TR implantation. The study was conducted in compliance with the HIPAA privacy practices and the tenets of the Declaration of Helsinki. Informed consent was obtained prior to the procedure. A waiver was granted for data analysis after ethics review (WCG IRB, Exemption Approval #1-1806618-1).

### 2.2. Implant and Implantation Procedure

The iDose TR delivery system consists of a biocompatible titanium implant preloaded in a single-use inserter. The implant measures 1.8 mm × 0.5 mm and comprises a drug reservoir containing 75 µg of preservative-free travoprost, a proprietary elution membrane, a scleral anchor, and a cap securing the membrane. The implant is introduced ab internally via a clear corneal incision, inserted through the trabecular meshwork, and anchored into the sclera at the iridocorneal angle. The implant and insertion procedure have been described in detail previously [9,13].

At the end of each procedure, intracameral moxifloxacin was injected and eyes were prescribed low-dose topical steroids 4 times daily for 4 days. All topical glaucoma medications were stopped postoperatively.

Prior to surgery, target IOP for each eye was defined as IOP reduced by ≥20% from baseline. Treatment was escalated if target IOP was not achieved postoperatively. The surgeon customarily performed MIGS for treatment escalation rather than reinitiating or adding topical medication(s).

### 2.3. Endpoints

Data were analyzed at baseline and at postoperative Months 1, 3, 6, and 12. “Baseline” was defined as the visit immediately before the procedure; no topical medication washout was completed prior to this visit. Efficacy endpoints were mean IOP (measured by Goldmann applanation tonometry); the proportions of eyes with IOPs ≤ 18 mmHg, ≤15 mmHg, and ≤12 mmHg; and the proportions of eyes on 0, 1, 2, or 3 topical medications (range = 0 to 3). Subgroup analyses were done for eyes grouped by history of SLT treatment (no prior SLT vs. prior SLT) and for eyes grouped by glaucoma severity according to the Hodapp–Parrish–Anderson criteria (mild, moderate, and severe) [14]. For the purpose of subgroup analysis, eyes with OHT were grouped with eyes with mild glaucoma to yield the mild/OHT subgroup. Safety endpoints included adverse events (intraoperative or postoperative), SSIs, visual field mean deviation (MD), cup-to-disc ratio, and retinal nerve fiber layer thickness (RNFL).

### 2.4. Statistical Analysis

Primary analyses were based on an intention-to-treat (ITT) principle. Eyes that required secondary surgical interventions (SSIs) to reach target IOP were not excluded from analyses according to the ITT approach. A sensitivity analysis was performed considering eyes requiring SSIs as treatment failures in order to validate the results of the primary analyses. Missing data were considered missing-at-random, and no imputation of missing values was performed. As this was a real-world study, no strategies were employed to increase compliance to follow-ups.

Statistical analysis was performed with Microsoft Excel (version 16) and SAS (version 9.4 M9) statistical software. Paired *t*-tests were used to compare baseline to 12-month continuous outcomes in the ITT cohort. Mixed-effects models were used to compare baseline to 12-month continuous outcomes in analyses of changes within and between subgroups. McNemar’s test was used to compare baseline to 12-month dichotomous outcomes in the ITT cohort. The Wilcoxon signed-rank test was used to compare baseline to 12-month medication count outcomes in the ITT cohort. Generalized linear mixed-effects models were used to compare baseline to 12-month medication count data in analyses of changes within and between subgroups. A *p*-value < 0.05 was considered statistically significant; all tests were 2-tailed.

The sensitivity analysis repeated the primary analysis using the ITT cohort with baseline imputation for patients who experienced treatment failure. For these patients, endpoint values were imputed on or after the time of SSI using patients’ baseline values.

Given an n of 65, with mean change of −5.98, baseline SD = 3.97, and a baseline and 12-month IOP Pearson’s r of 0.246, the study had a power greater than 99.9% to detect a 12-month change from the baseline for IOP. Given an n of 65, with a mean change of −0.02, a baseline SD = 0.66, and a baseline and 12-month IOP Pearson’s r of 0.542, the study had a power of 5.4% to detect a 12-month change from the baseline for medical burden.

## 3. Results

### 3.1. Demographics

The study included 65 eyes of 37 patients (mean age: 79.7 ± 7.1 years) (Table 1). Ten eyes underwent SSI during the 12-month follow-up, according to the surgeon’s standard practice of using MIGS procedures rather than reinitiating or adding medication(s) if target IOP was not attained; these eyes were not excluded from the primary analyses as per the intention-to-treat approach. All eyes were pseudophakic and underwent standalone iDose TR implantation. As the surgeon customarily practices interventional glaucoma treatment to reduce topical medication burden, 100% of the eyes had undergone prior glaucoma procedure(s), which are listed in Table 1.

### 3.2. Efficacy Outcomes

**Figure 1:**
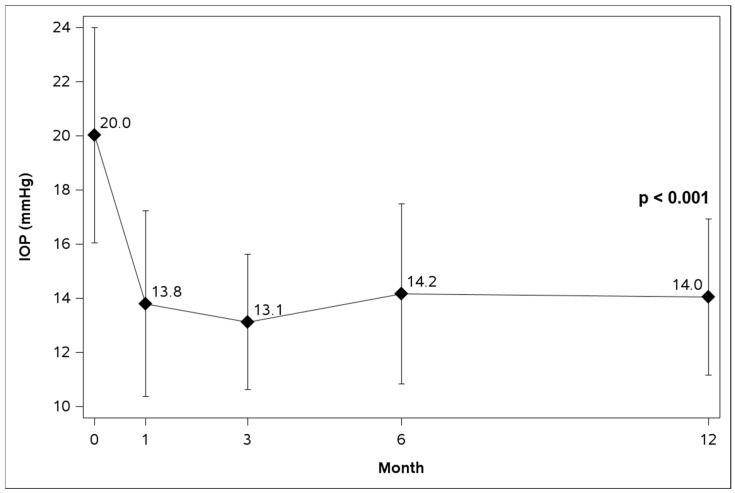
Mean intraocular pressure (IOP) over time in the overall ITT cohort (n = 65).

**Figure 2:**
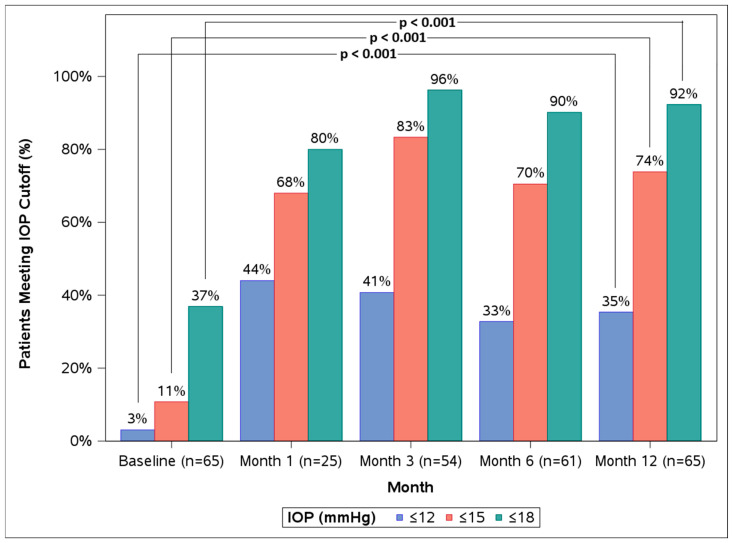
Baseline vs. 12-month (12 M) proportions of eyes with IOP ≤ 18 mmHg, ≤15 mmHg, and ≤12 mmHg in the overall ITT cohort.

In the overall ITT cohort, mean IOP significantly reduced from 20.0 ± 4.0 mmHg at baseline to 14.0 ± 2.9 mmHg at 12 months (28% reduction, *p* < 0.001) (Table 2, Figure 1). The proportions of eyes with IOPs ≤ 18 mmHg, ≤15 mmHg, and ≤12 mmHg were 36.9%, 10.8%, and 3.1%, respectively, at baseline, which increased to 92.3%, 73.8%, and 35.4%, respectively, at 12 months (*p* < 0.001 for all) (Table 2, Figure 2). The proportions of eyes on 0, 1, 2, and 3 topical medications were not significantly altered from baseline to 12 months (*p* = 0.853) (Table 3).

**Figure 3:**
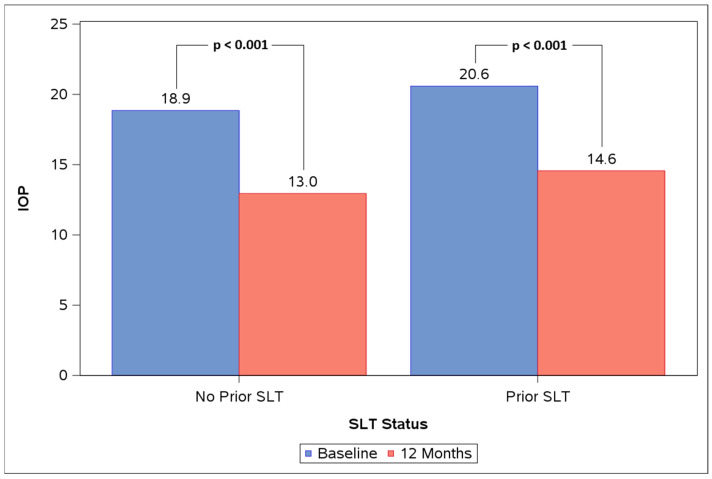
Baseline vs. 12 M mean IOP in the no-prior-selective laser trabeculoplasty (SLT) and prior-SLT subgroups.

Both the no-prior-SLT and prior-SLT subgroups experienced significant IOP reductions at 12 months vs. baseline (Figure 3, Table 4). In the no-prior-SLT group (n = 21), mean IOP reduced from 18.9 ± 2.3 mmHg to 13.0 ± 1.8 mmHg at 12 months (31% reduction, *p* < 0.001). In the prior-SLT group (n = 44), mean IOP reduced from 20.6 ± 4.5 mmHg to 14.6 ± 3.2 mmHg (26% reduction, *p* < 0.001). The difference in IOP change from baseline to 12 months between subgroups was not significant (*p* = 0.907). The change in proportions of eyes on 0, 1, 2, and 3 topical medications from baseline to 12 months was not significant within either subgroup (no-prior-SLT *p* = 0.848, prior-SLT *p* = 0.995). The difference between subgroups in terms of the change in proportions of eyes on 0, 1, 2, and 3 topical medications from baseline to 12 months was not significant (*p* = 0.907).

**Figure 4:**
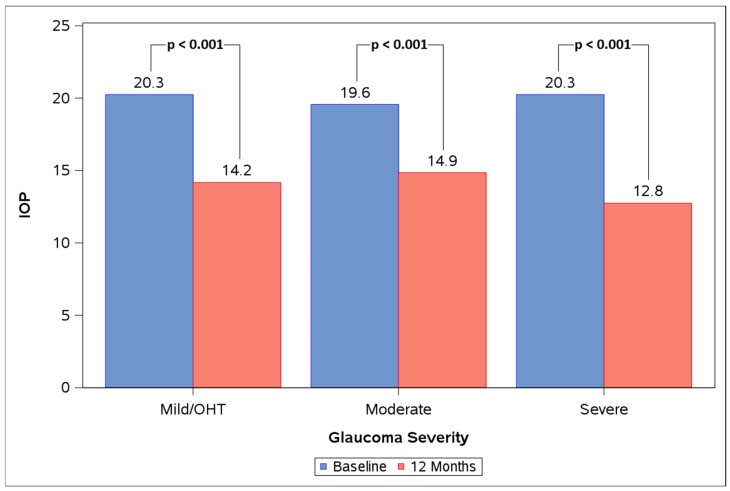
Baseline vs. 12 M mean IOP in the mild/ocular hypertension (OHT), moderate, and severe subgroups.

The mild/OHT, moderate, and severe subgroups all experienced significant IOP reductions at 12 months vs. baseline (Figure 4, Table 5). Mean IOP reduced from 20.3 ± 4.1 mmHg at baseline to 14.2 ± 2.7 mmHg at 12 months in the mild/OHT group (n = 28), from 19.6 ± 3.0 mmHg at baseline to 14.9 ± 3.0 mmHg at 12 months in the moderate group (n = 21), and from 20.3 ± 4.9 mmHg at baseline to 12.8 ± 2.8 mmHg at 12 months in the severe group (n = 16) (28%, 23%, and 34% reductions, respectively; *p* < 0.001 for all). The difference in mean IOP change from baseline to 12 months between subgroups was not significant (*p* = 0.085). The change in proportions of eyes on 0, 1, 2, and 3 topical medications from baseline to 12 months was not significant within any subgroup (mild/OHT *p* = 0.991, moderate *p* = 0.486, severe *p* = 0.819). The difference between subgroups in the change in proportions of eyes on 0, 1, 2, and 3 topical medications from baseline to 12 months was not significant (*p* = 0.775).

### 3.3. Safety Outcomes

In the overall cohort (n = 65), visual field MD reduced from −5.59 ± 7.74 dB at baseline to −6.83 ± 7.82 dB at 12 months (*p* = 0.043). No statistically significant changes were observed from baseline to 12 months in the cup-to-disc ratio (0.77 ± 0.10 vs. 0.77 ± 0.10; *p* = 0.999) and RNFL thickness (72.3 ± 14.4 µm to 71.1 ± 15.3 µm; *p* = 0.238).

No intraoperative complications were observed in any eye. Postoperative complications were noted in two eyes (3.1%): corneal edema was observed at Week 1 in one eye (moderate glaucoma, no prior SLT), while retinal edema was observed at Month 6 in one eye (moderate glaucoma, prior SLT). Both were deemed to be “possibly” implant-related. Both complications were transient and resolved by the subsequent visit without intervention or sequelae. There were no cases of conjunctival hyperemia, hypertrichosis of eyelashes, or periorbital fat atrophy. No eyes had serious corneal adverse events or required implant removal over the follow-up period.

### 3.4. Eyes with Secondary Intervention

In total, 10 eyes underwent SSI during the study duration (mild: 1, moderate: 5, severe: 4), according to the surgeon’s standard practice of using minimally invasive procedures rather than adding medication(s) in patients who did not attain target IOP. In five eyes, SSIs were performed despite a ≥20% IOP reduction from baseline as they did not achieve target IOP according to their respective glaucoma severities. In the remaining eyes, SSIs were performed because IOP was either marginally reduced (n = 2), unchanged (n = 2) or increased (n = 1) compared to baseline. One eye underwent iStent infinite implantation at 3 months and three eyes underwent iStent infinite implantation, while one eye underwent iStent infinite implantation combined with cyclophotocoagulation (CPC) at 6 months and five eyes underwent iStent infinite implantation at 12 months. In one eye that had undergone iStent infinite surgery at 6 months, IOP remained uncontrolled post-SSI, due to which CPC was performed at 12 months.

### 3.5. Sensitivity Analysis

When eyes requiring SSIs were considered as treatment failures, mean IOP showed a significant reduction from 20.0 ± 4.0 mmHg preoperatively to 14.7 ± 3.7 mmHg at 12 months (*p* < 0.001) (Table 6). The proportions of eyes with IOP ≤ 18 mmHg, ≤15 mmHg, and ≤12 mmHg were 36.9%, 10.8%, and 3.1%, respectively, at baseline, which increased to 86.2%, 69.2%, and 32.3%, respectively, at 12 months (*p* < 0.001). The proportions of eyes on 0, 1, 2, and 3 topical medications were not significantly altered from baseline to 12 months (*p* = 0.707).

## 4. Discussion

Intracameral prostaglandin implants are a relatively recent addition to the glaucoma treatment algorithm following their performance vs. topical glaucoma medication in pivotal trials [11,13,15,16]. Real-world data on these implants are limited due to their relatively short history in clinical use. The current real-world study evaluates the iDose TR travoprost implant in a routine practice setting and adds to the data on its long-term efficacy and safety. To the authors’ knowledge, this is the first study on real-world outcomes with the iDose TR in patients with OAG and OHT over 12 months.

In this study, the travoprost implant significantly reduced IOP by 28% at 12 months from a baseline of 20.0 ± 4.0 mmHg, with a majority of eyes achieving IOP ≤ 15 mmHg (73.8%). The low medication burden observed preoperatively was maintained throughout the study period, with no significant change in proportions of eyes on 0, 1, 2, and 3 topical medications from baseline to 12 months. The sensitivity analysis observed a significant mean IOP reduction to the mid-teens, a significant increase in the proportions of eyes reaching prespecified IOP thresholds, and no significant change in the proportions of eyes on 0, 1, 2, and 3 topical medications, which aligned with the results of the primary analysis. In the subgroup analysis, no statistically significant differences were noted for the degree of IOP reduction between eyes with or without a history of SLT treatment, or between eyes with mild/OHT, moderate, or severe glaucoma. Mild, transient, self-resolving complications were observed in two eyes (3.1%).

The mean IOP reduction of approximately 6.0 mmHg at 12 months observed in this study is lower than that noted in Phase III trials at 12 months for the travoprost implant (6.8 mmHg–8.5 mmHg) [11]. This is as expected given that the pivotal trials performed preoperative medication washouts and thus observed higher mean baseline IOPs (~24 mmHg), while medication washout was not performed in the current study due to its inappropriateness in the real-world study population, resulting in a lower baseline IOP (20 mmHg). A higher baseline IOP is known to be associated with greater absolute and relative IOP reduction, which can explain the higher IOP-lowering efficacy of the iDose TR in the pivotal trials compared to the current study.

The preoperative mean medication burden was lower in the current study (0.23 ± 0.66 vs. 0.9 ± 0.8 in the Phase III trials), with a higher proportion of medication-free eyes at baseline (87.7% vs. 33.2% in the Phase III trials). This is likely because all eyes in the current study had undergone prior glaucoma procedures to lower topical medication burden as per the interventional glaucoma approach followed by the primary surgeon. The travoprost implant was able to reduce IOP while further increasing the proportion of medication-free eyes from 87.7% preoperatively to 89.2% at 12 months, which value was roughly comparable to that of 81.4% for medication-free eyes observed at the same timepoint in the Phase III trials [11].

Formal registration-facing clinical trials are crucial in providing definitive and robust evidence, while studies on real-world usage in diverse populations and routine settings must also be evaluated, as they provide valuable information on the clinical utility of a procedure in actual use. To date, this is the first 12-month study to report real-world outcomes with the travoprost implant, but comparisons can be made with 12-month real-world studies on single bimatoprost intracameral implant administrations. For example, in a retrospective study by Ali et al. [17], eyes without subsequent IOP-lowering interventions (medication or surgery) showed a marginal increase in IOP by 1.3 mmHg at 12 months from a baseline of 14.5 ± 3.6 mmHg (*p* = 0.047). In the overall cohort, medications were reduced by 0.5 at 12 months from a baseline of 1.7 ± 0.9, with 27.8% of eyes being medication-free compared to 8.5% preoperatively. In another retrospective study [18], the bimatoprost implant reduced IOP and medication burden from 16.6 mmHg and 1.4 medications at baseline to 13.3 mmHg and 0.2 medications over 11–13 months of follow-up. Topical medication-free eyes increased from 5.1% at baseline to 89.9% postoperatively. Comparatively, the present study showed greater IOP reduction and similar or higher proportions of topical-medication-free eyes at 12 months.

The current study also analyzed the effectiveness of the travoprost implant in eyes grouped by a history of SLT treatment and by glaucoma severity. Eyes with or without prior SLT exhibited a similar degree of IOP reduction at 12 months vs. baseline (−6.0 mmHg vs. −5.9 mmHg, *p* = 0.907). Similarly, eyes with mild/OHT, moderate, or severe glaucoma showed no significant differences in 12-month IOP reductions (−6.1 mmHg, −4.7 mmHg, and −7.5 mmHg, respectively; *p* = 0.085). The cohort’s inclusion of severe glaucoma is an especially important addition to the literature, as it reinforces the mild to moderate data from the Phase 2 and Phase 3 clinical trials. Our findings indicate that the travoprost implant is efficacious regardless of history of prior SLT or glaucoma severity.

The travoprost intracameral implant demonstrated a favorable safety profile, with no serious corneal adverse events in pivotal trials, validating the implant design principle that anchors the implant in the sclera, thereby preventing corneal endothelial injury due to implant movement in the anterior chamber [9,11]. A similarly high safety profile was observed in the current study, with no intraoperative complications and only two postoperative adverse events, which were transient and resolved without intervention or sequelae by the subsequent visit. Complications associated with topical prostaglandin use, such as ocular surface disorders, hypertrichosis of eyelashes, periorbital pigmentation, or periorbital fat atrophy were not observed in the pivotal trials [9,11,13] or in the current study. The lack of prostaglandin-associated adverse events in the ocular surface and adnexa may demonstrate the benefit of targeted intracameral drug delivery, applying active medication directly to the target tissues [19]. The structural parameters of RNFL thickness and cup-to-disc ratio showed no significant changes from baseline to 12 months. The reduction in mean visual field MD observed in the current study (−1.24 dB) should be considered with caution due to the retrospective context of the study. Visual field interpretation should also keep in mind the need for intracameral travoprost implant administration despite prior glaucoma procedure(s) in all eyes and the higher age of the cohort (mean: 79.7 ± 7.1 years), which has been associated with more rapid visual field progression.

This study is limited by its retrospective, single-center design and modest sample size. The study was sufficiently powered to detect a change in IOP (power >99.9%); however, it was insufficiently powered (power of ~5%) to detect a change in medical burden. Thus, the null finding for a change in medical burden could be due to the lack of statistical power, the data for this study not being sufficiently powered to allow this question to be answered. Additionally, the study was not adequately powered to detect differences in effectiveness between subgroups. Further studies with larger cohorts could answer these questions with sufficient sample sizes. All patients were pseudophakic and had a history of prior glaucoma procedures due to the surgeon’s interventional glaucoma treatment approach, which could reduce the generalizability of our findings in phakic or treatment-naïve patients. The retrospective design and single-center setting may also limit generalizability. To minimize possible confounding of IOP-lowering efficacy by prior glaucoma procedure(s), the study only included eyes with prior interventions performed >6 months before travoprost implant administration. Some degree of selection bias could have been introduced due to loss to follow-up and missing data.

Despite these limitations, this study provides valuable information on the performance of the travoprost implant in this real-world population due to the inclusion of the surgeon’s actual patient base. Additionally, the primary intention-to-treat analyses and the reporting of data without imputation for missing observations reduced bias compared to a per-protocol approach, preventing overestimation of implant effectiveness. The consistency between the primary ITT analysis and the sensitivity analysis results further validate the robustness of our findings while accounting for confounding due to secondary intervention(s). Follow-up is ongoing for the cohort included in this study, allowing longer-term outcomes to be evaluated in the future.

## 5. Conclusions

In conclusion, in this study, standalone implantation of a single iDose TR travoprost intracameral implant in patients with open-angle glaucoma and ocular hypertension previously treated with SLT, bimatoprost intracameral implant, or MIGS demonstrated favorable efficacy and safety over 12 months post-procedure. IOP was significantly reduced at 12 months, with a concomitant increase in the proportion of topical-medication-free eyes. IOP reductions in the cohort were significant regardless of a history of SLT treatment and across all glaucoma severities, with no statistically significant differences between eyes with or without prior SLT or between mild/OHT, moderate, and severe glaucoma eyes. Meanwhile, structural parameters like RNFL thickness and the cup-to-disc ratio remained stable over 12 months, and postoperative adverse events were minimal and self-resolving. The findings of this study demonstrate that an interventional glaucoma treatment approach using the travoprost intracameral implant can effectively and safely provide long-term IOP control in a real-world clinical setting and that these benefits are achieved regardless of prior SLT status or glaucoma severity.

## Figures and Tables

**Table 1:** Demographics and preoperative measurements of the overall ITT cohort.

Patients	N = 37
**Age, years (mean ± SD (min, max))**	79.7 ± 7.1 (68.8, 94.8)
**Gender (n (%))**	
Male	17 (45.9%)
Female	20 (54.1%)
**Ethnicity (n (%))**	
Asian	1 (2.7%)
Hispanic	1 (2.7%)
White	33 (89.2%)
Missing	2 (5.4%)
**Eyes**	**N = 65**
**Lens status (n (%))**	
Phakic	0 (0.0%)
Pseudophakic	65 (100.0%)
**Diagnosis (n (%))**	
OHT	3 (4.6%)
Mild POAG	25 (38.5%)
Moderate POAG	21 (32.3%)
Severe POAG	16 (24.6%)
**Prior glaucoma procedure(s) (n (%))**	
Yes	65 (100.0%)
No	0 (0.0%)
Prior glaucoma procedures (n (%)) *	
Durysta	61 (93.8%)
SLT	44 (67.7%)
CPC	7 (10.8%)
iStent	2 (3.1%)
KDB goniotomy	1 (1.5%)
**Preoperative measurements (mean ± SD (min, max))**	
Cup-to-disc ratio	0.77 ± 0.10 (0.35, 0.95)
Visual field mean deviation (dB)	−5.59 ± 7.74 (−30.29, 11.68)
RNFL thickness (µm)	72.3 ± 14.4 (26, 107)
Baseline medications	0.23 ± 0.66 (0, 3)
Medication burden (n (%))	
0	57 (87.7%)
1	2 (3.1%)
2	5 (7.7%)
3	1 (1.5%)
Baseline IOP	20.0 ± 4.0 (11, 30)
IOP distribution (n (%))	
IOP ≤ 18 mmHg	24 (36.9%)
IOP ≤ 15 mmHg	7 (10.8%)
IOP ≤ 12 mmHg	2 (3.1%)

* Eyes could have more than 1 prior procedure. SD, standard deviation; min, minimum; max, maximum; OHT, ocular hypertension; POAG, primary open-angle glaucoma; SLT, selective laser trabeculoplasty; CPC, cyclophotocoagulation; KDB, Kahook Dual Blade; RNFL, retinal nerve fiber layer; IOP, intraocular pressure.

**Table 2:** IOP outcomes in the overall ITT cohort at all timepoints.

	Baseline (n = 65)	Month 1 (n = 25)	Month 3 (n = 54)	Month 6 (n = 61)	Month 12 (n = 65)	*p*-Value (12 M vs. Baseline)
**IOP, mmHg** (mean ± SD)	20.0 ± 4.0	13.8 ± 3.4	13.1 ± 2.5	14.2 ± 3.3	14.0 ± 2.9	<0.001 *
**IOP distribution (n (%))**						
IOP ≤ 18 mmHg	24 (36.9%)	20 (80.0%)	52 (96.3%)	55 (90.2%)	60 (92.3%)	<0.001 *
IOP ≤ 15 mmHg	7 (10.8%)	17 (68.0%)	45 (83.3%)	43 (70.5%)	48 (73.8%)	<0.001 *
IOP ≤ 12 mmHg	2 (3.1%)	11 (44.0%)	22 (40.7%)	20 (32.8%)	23 (35.4%)	<0.001 *

* Statistically significant. IOP, intraocular pressure; SD, standard deviation.

**Table 3:** Proportional analysis of medication burden in the overall ITT cohort at all timepoints.

Medication Burden (n (%))	Baseline (n = 65)	Month 1 (n = 25)	Month 3 (n = 55)	Month 6 (n = 61)	Month 12 (n = 65)	*p*-Values (12 M vs. Baseline)
0	57 (87.7%)	25 (100.0%)	55 (100.0%)	54 (88.5%)	58 (89.2%)	
1	2 (3.1%)	0 (0.0%)	0 (0.0%)	5 (8.2%)	1 (1.5%)	
2	5 (7.7%)	0 (0.0%)	0 (0.0%)	1 (1.6%)	5 (7.7%)	
3	1 (1.5%)	0 (0.0%)	0 (0.0%)	1 (1.6%)	1 (1.5%)	
*p*-value for medication counts (12 M vs. Baseline)						0.853

**Table 4:** Mean IOP in eyes grouped by SLT history (no prior SLT, prior SLT).

	Baseline	Month 1	Month 3	Month 6	Month 12	*p*-Value (12 M vs. Baseline)
**No prior SLT**						
IOP, mmHg (mean ± SD)	18.9 ± 2.3 (n = 21)	11.7 ± 1.2 (n = 6)	12.3 ± 2.2 (n = 18)	12.5 ± 2.5 (n = 19)	13.0 ± 1.8 (n = 21)	<0.001 *
**Prior SLT**						
IOP, mmHg (mean ± SD)	20.6 ± 4.5 (n = 44)	14.5 ± 3.6 (n = 19)	13.6 ± 2.6 (n = 36)	14.9 ± 3.4 (n = 42)	14.6 ± 3.2 (n = 44)	<0.001 *
*p*-value between subgroups (12-month IOP reduction)						0.907

* Statistically significant. IOP, intraocular pressure; SLT, selective laser trabeculoplasty; SD, standard deviation.

**Table 5:** Mean IOP in eyes grouped by glaucoma stage (mild/OHT, moderate, severe).

	Baseline	Month 1	Month 3	Month 6	Month 12	*p*-Value (12 M vs. Baseline)
**Mild/OHT**						
IOP, mmHg (mean ± SD)	20.3 ± 4.1 (n = 28)	14.4 ± 4.2 (n = 10)	13.3 ± 2.5 (n = 23)	14.5 ± 3.6 (n = 28)	14.2 ± 2.7 (n = 28)	<0.001 *
**Moderate**						
IOP, mmHg (mean ± SD)	19.6 ± 3.0 (n = 21)	13.0 ± 1.9 (n = 9)	12.7 ± 1.9 (n = 20)	14.3 ± 2.8 (n = 18)	14.9 ± 3.0 (n = 21)	<0.001 *
**Severe**						
IOP, mmHg (mean ± SD)	20.3 ± 4.9 (n = 16)	14 ± 4.1 (n = 6)	13.6 ± 3.4 (n = 11)	13.3 ± 3.5 (n = 15)	12.8 ± 2.8 (n = 16)	<0.001 *
*p*-value between subgroups (12-month IOP reduction)						0.085

* Statistically significant. IOP, intraocular pressure; OHT, ocular hypertension; SD, standard deviation.

**Table 6:** Sensitivity analysis considering eyes that underwent SSI as treatment failures.

	Baseline (n = 65)	Month 1 (n = 25)	Month 3 (n = 55)	Month 6 (n = 61)	Month 12 (n = 65)	*p*-Value (12 M vs. Baseline)
**IOP, mmHg** (mean ± SD)	20.0 ± 4.0	13.8 ± 3.4	13.1 ± 2.5	14.4 ± 3.6	14.7 ± 3.7	<0.001 *
**IOP distribution (n (%))**						
IOP ≤ 18 mmHg	24 (36.9%)	20 (80%)	52 (96.3%)	54 (88.5%)	56 (86.2%)	<0.001 *
IOP ≤ 15 mmHg	7 (10.8%)	17 (68%)	45 (83.3%)	42 (68.9%)	45 (69.2%)	<0.001 *
IOP ≤ 12 mmHg	2 (3.1%)	11 (44%)	22 (40.7%)	19 (31.1%)	21 (32.3%)	<0.001 *
**Medication** **burden (n (%))**						
0	57 (87.7%)	25 (100.0%)	55 (100.0%)	54 (88.5%)	57 (87.7%)	
1	2 (3.1%)	0 (0.0%)	0 (0.0%)	5 (8.2%)	3 (4.6%)	
2	5 (7.7%)	0 (0.0%)	0 (0.0%)	1 (1.6%)	5 (7.7%)	
3	1 (1.5%)	0 (0.0%)	0 (0.0%)	1 (1.6%)	0 (0.0%)	
*p*-value for medication counts (12 M vs. Baseline)						0.707

* Statistically significant. SSI, secondary surgical intervention; IOP, intraocular pressure; SD, standard deviation.

## Data Availability

The datasets generated during and/or analyzed during the current study are not publicly available due to patient privacy guidelines.
